# Ovarian tissue transplantation: 10 years of experience at the Bologna University

**DOI:** 10.3389/fendo.2024.1332673

**Published:** 2024-03-07

**Authors:** Raffaella Fabbri, Rossella Vicenti, Valentina Magnani, Roberto Paradisi, Lucia De Meis, Diego Raimondo, Alessandro Arena, Stefano Venturoli, Antonio Raffone, Arianna Raspollini, Renato Seracchioli

**Affiliations:** ^1^ Department of Medical and Surgical Sciences, University of Bologna, Bologna, Italy; ^2^ Division of Gynaecology and Human Reproduction Physiopathology, IRCCS Azienda Ospedaliero-Universitaria di Bologna, Bologna, Italy

**Keywords:** ovarian tissue cryopreservation, fertility preservation, cancer, orthotopic and heterotopic transplantation, restoration of endocrine function, longevity, pregnancies, live births

## Abstract

**Objective:**

The efficiency of ovarian tissue transplantation (OTT) was established in terms of ovarian function recovery (95% of cases), number of live births (over 200 worldwide to date) and induction of puberty. Unfortunately, the lack of international registries and the fact that many centers have not yet reported their outcomes, lead to poor knowledge of the exact fertility data. The aim of the study is to describe our experience with OTT to restore ovarian function and fertility.

**Methods:**

This study was designed as a single-center, observational, retrospective, cohort study that includes women who underwent OTT between December 2012 and June 2023 at our center. After approval by the oncologist/hematologist, a small fragment of ovarian tissue was thawed and analyzed to detect the presence of micrometastases before OTT. Thawed ovarian tissue was grafted laparoscopically at multiple sites, including the remaining ovary and pelvic side wall (orthotopic transplantation) and/or abdominal wall (heterotopic transplantation). After OTT, ovarian function was monitored by hormonal assay, ultrasound and color Doppler at approximately 4-week intervals.

**Results:**

Between December 2012 and June 2023, 30 women performed OTT. Prior to OTT, immunohistochemical and molecular analyses revealed no micrometastases in all thawed ovarian tissue samples. In our series of 30 women, 20 of women were on premature ovarian insufficiency (POI), and the remaining ten cases still had oligomenorrhea and difficulty getting pregnant. Among the women with POI before OTT and at least 6 months follow-up, recovery of endocrine function was observed in all but one woman who underwent orthotopic transplantation (13 of 14 cases), in one out of two women who underwent both orthotopic and heterotopic transplantation (1 of 2 cases) and in all women who underwent heterotopic transplantation (4 of 4 cases). Women who underwent OTT to enhance fertility had no alterations in menstrual cycle and hormonal levels. In total, ten pregnancies were obtained in 25 women, resulting in four live births, two ongoing pregnancies and four spontaneous abortions.

**Conclusion:**

Our data can help patients and physicians in their discussions and decisions about the need and possibilities of preserving fertility.

## Introduction

Recent advances in cancer diagnosis, and the introduction of new chemo/radiotherapy protocols have significantly increased the survival rates of children and young women with cancer (1, 2). However, these treatments are gonadotoxic and can severely affect or totally destroy reproductive potential of women leading to premature ovarian insufficiency (POI) (3).

This risk varies from under 10% to over 95% depending on several factors: woman’s age, pre-treatment ovarian reserve, type and cumulative dose of chemotherapy (4). The onset of premature menopause induces side effects such as osteoporosis, cardiovascular disease and psychosexual dysfunction. In prepubertal girls also anticancer treatments can cause the absence of the first menstruation, growth retardation, lack of development of secondary sexual characteristics and psychosocial difficulties due to the comparison with peers (5). The increased life expectancy given to those women by the use of anticancer treatments can only be considered a success if their quality of life is effectively preserved. Recent studies have shown that the potential iatrogenic loss of fertility has a profound impact on young women and can be more stressful than the cancer diagnosis itself (6).

Ovarian tissue cryopreservation (OTC) is a valid strategy to preserve endocrine and reproductive function in pre and postpubertal women at high risk of POI caused by gonadotoxic treatments. At cancer remission, cryopreserved ovarian tissue can be reimplanted, allowing the recovery of ovarian function and spontaneous pregnancy. Since the first report of successful restoration of ovarian function after orthotopic transplantation of frozen-banked ovarian tissue in 2000, the first live birth in 2004 and the second in 2005 greatly accelerated the implementation of this fertility preservation method (7–9). Over the years, the efficiency of ovarian tissue transplantation (OTT) has been established in terms of ovarian function recovery (95% of cases), number of live births (over 200 worldwide reported to date) and induction of puberty (1, 2, 10–12). Unfortunately, the lack of international registries, the fact that many centers have not yet reported their outcomes and the presence of data based on case reports from specialized centers, lead to poor knowledge of the exact fertility data in women after OTT (13). Recently some meta-analyzes have been conducted to analyze the data on fertility outcomes after OTT including pregnancy, live birth, miscarriage rates, and endocrine outcomes such as estrogen, Follicle Stimulating Hormone (FSH) and Luteinizing hormone (LH) levels. These analyses demonstrated that OTT could restore reproductive and hormonal functions in women submitted to the procedure (14–18).

The aim of the study is to describe the 10 years experience of our center with OTT to restore ovarian function and fertility.

## Material and methods

### Study protocol and subjects

This study was designed as a single-center, observational, retrospective, cohort study according to a prior defined study protocol. We reviewed clinical records and databases for all consecutive women who underwent OTT between December 2012 and June 2023 at our center.

The OTT was recommended for women who had cryopreserved their ovarian tissue for malignant or benign diseases and who went on POI or those, who did not go on POI, who did not have a spontaneous pregnancy. In addition, OTT was recommended for women interested in restoring endocrine function instead of having a specific desire for motherhood. Other eligibility criteria for OTT were: oncologist/hematologist approval, absence of neoplastic contamination in cryopreserved ovarian tissue and no contraindication to laparoscopy. The OTT procedures were approved by our local Ethics Committee (N. 74/2001/O). An informed written consent was signed by all women.

### Ovarian tissue cryopreservation (OTC)

A large ovarian biopsy of approximately 40% of one or both ovaries was obtained according to the procedure of Paradisi et al. (19). For young girls an ovarian biopsy from one or both ovaries was obtained by laparoscopy according to the procedure of Lima et al. (20). The cortical tissue was dissected into strips (mean size of 1 cm × 2 mm × 1 mm), and slowly frozen according to the protocol described by Fabbri et al. (21). In brief, ovarian cortical strips were placed in plastic cryovials (Intermed Nunc Cryotubes, Roskilde, Denmark) containing 1.8 ml of “freezing solution” consisting of 1.5 M 1,2-propanediol (PROH - Fluka Chemica, Sigma Aldrich SrL; Milan, Italy), 0.2 M sucrose (Fluka Chemica, Sigma Aldrich SrL; Milan, Italy), and 30% heat-inactivated human serum (HS - provided by the Transfusion Centre of S. Orsola Malpighi Hospital) in Dulbecco’s phosphate-buffered saline (DPBS-Gibco, Life Technologies, Paisley, Scotland) and maintained at 4°C in an ice bath. The cryovials were transferred to a rolling system (Continents instrument, Amityville, USA) for 30 min at 4°C to allow the cryoprotectant to enter the tissue; they were then cooled in an programmable freezer (Planer Kryo 10/1,7 Series III, SAPIO Life, Milan, Italy) allowing the gradual reduction of the temperature from 0 to −140°C. The starting temperature was 0°C; then it was slowly reduced to −9°C at a rate of 2°C/min. Ice nucleation was induced manually at −9°C (seeding). After a holding time of 10 min at −9°C, the cryovials were cooled slowly to −40°C at a rate of 0.3°C/min and then rapidly to −140°C at a rate of 10°C/min. After 10 min of temperature stabilization, the cryovials were transferred into liquid nitrogen tanks and stored until thawing. Frozen ovarian tissue was thawed using a modified rapid-thawing protocol described by Fabbri et al. (22). In brief, the cryovials were air-warmed for 30 s and then immersed into 37°C water bath for 2 min. The cryoprotectants were removed at 4°C by four-stepwise dilution: (i) 0.76 M PROH, 0.175 M sucrose, 30% HS in DPBS for 5 min; (ii) 0.26 M PROH, 0.175 M sucrose, 30% HS in DPBS for an additional 5 min; (iii) 0.175 M sucrose, 30% HS in DPBS for 10 min and finally, (iv) DPBS supplemented with 30% HS for 20 min.

### Analysis of fresh and thawed ovarian tissue

Histological analysis was performed on fresh and thawed samples to assess the follicular development stage according to Gougeon (23), the follicular density (number of follicles per mm^2^ of the overall section area), the morphological characteristics of the follicles and stromal cells. Immunohistochemical and molecular analyses were also performed on thawed samples to detect the presence of ovarian tissue micrometastases, as previously described (24–26).

One fragment (± 2 mm × 2 mm × 1 mm) from each ovary was fixed at the time of laparoscopy (fresh tissue) and after thawing in 4% formaldehyde, embedded in paraffin and serially sectioned at a thickness of 4 μm. Six serial sections were obtained per block: the first, third and fifth were stained with hematoxylin and eosin (Merck, Darmstadt, Germany) to assess the presence of malignant cells in the ovarian tissue and the morphological features of follicles and stromal cells. The second, fourth and sixth sections were incubated with different antibodies for immunohistochemistry to detect the presence of micrometastasis in the ovarian tissue. Molecular analysis was performed using Real-time PCR and PCR assay with EUROCLONALITY protocols evaluated by GeneScanning analysis to determine clonal character, according to Fabbri et al. (26, 27).

### Ovarian tissue transplantation

The OTT was carried out after a remission period of at least 2 years, following the approval of the oncologist/hematologist.

**Figure 1:**
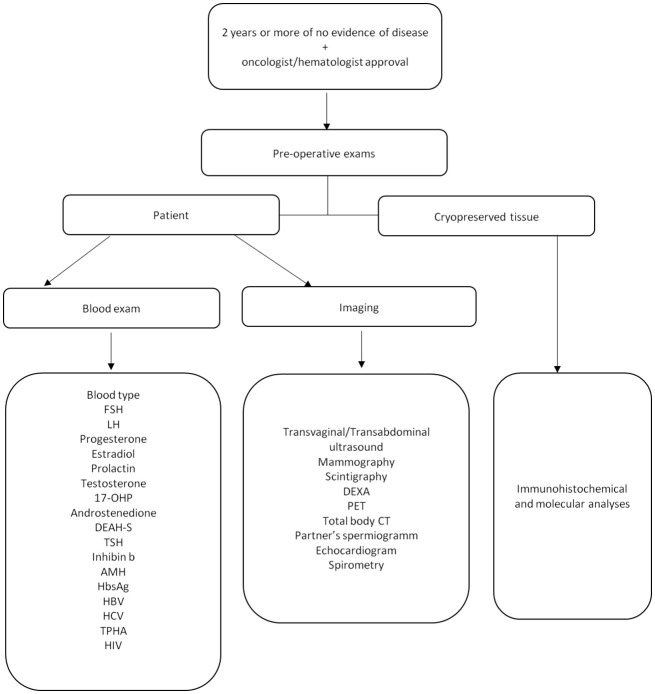
Pre-operative period flow chart. Follicle-stimulating Hormone (FSH), luteinizing hormone (LH), 17-hydroxyprogestrone (17-OHP), dehydroepiandrosterone sulfate (DEAH-S), thyrotropin (TSH), Anti-Mullerian Hormone (AMH), Hepatitis B surface antigen (HBs Ag), hepatitis B virus (HBV), hepatitis C virus (HCV), Treponema pallidum (TPHA), human immunodeficiency virus (HIV), dual-energy-xray-absorptiometry (DEXA), positron emission tomography (PET), total-body computed tomography (Total body, CT).

Before proceeding with the OTT all women underwent several examinations including computed axial tomography, densitometry and mammography in order to evaluate general health. Moreover, the couple was screened for other potential causes of infertility therefore the patency of the Fallopian tubes by sonosalpingography, an evaluation of the uterus by hysteroscopy were performed (**Figure 1**) for women and semen analysis was performed for the partner.

Before OTT, a small fragment (2 mm × 2 mm × 1 mm) of ovarian tissue was thawed and analyzed to detect the presence of micrometastases using immunohistochemical and molecular analyses (**Figure 1**).

Thawed ovarian tissue was grafted laparoscopically at multiple sites, including the remaining ovary and pelvic side wall (orthotopic transplantation) and/or abdominal wall (heterotopic transplantation). The graft sites used were largely determined at the time of the operation, depending on the women’s desire (fertility reactivation or only endocrine function restoring), pelvic anatomy (residual ovarian volume and the presence of previous adhesions) and past treatments (pelvic radiotherapy).

**Figure 2:**
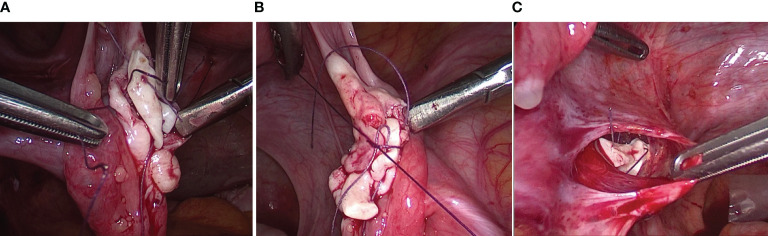
Surgical techniques of orthotopic transplantation of cryopreserved ovarian tissue. **(A, B)** A longitudinal incision was made on the surface of ovaries and a pocket was developed in the ovarian parenchyma by blunt dissection. Cortical strips were sutured into the ovarian pocket and the same sutures were used to close the ovary. **(A)** Transplanted right ovary (3 samples with following sizes 1.5 cm × 4 mm × 1 mm); **(B)** Transplanted left ovary (2 samples with following sizes 1.2 cm × 3 mm × 1 mm). **(C)** An incision was made on the surface of the peritoneum near the ovarian vessels and fimbria. Cortical strips were inserted into the pocket and a 4-0 Vicryl suture were used to close the peritoneal pocket (2 samples with following sizes 1 cm × 4 mm × 1 mm).

The orthotopic and heterotopic transplantation procedures were described in Fabbri et al. (24). Orthotopic transplantation was performed by four-port laparoscopy. A longitudinal incision of approximately 1 cm was made on the surface of ovaries and a pocket was developed in the ovarian parenchyma by blunt dissection. Cortical strips were sutured into the ovarian pocket and the same sutures were used to close the ovary, taking care not to cause tissue ischemia. To increase the chances of ovarian function recovery, a peritoneal pocket was also created near the ovarian vessels and fimbria, and closed with a 4-0 Vicryl suture (**Figure 2**).

**Figure 3:**
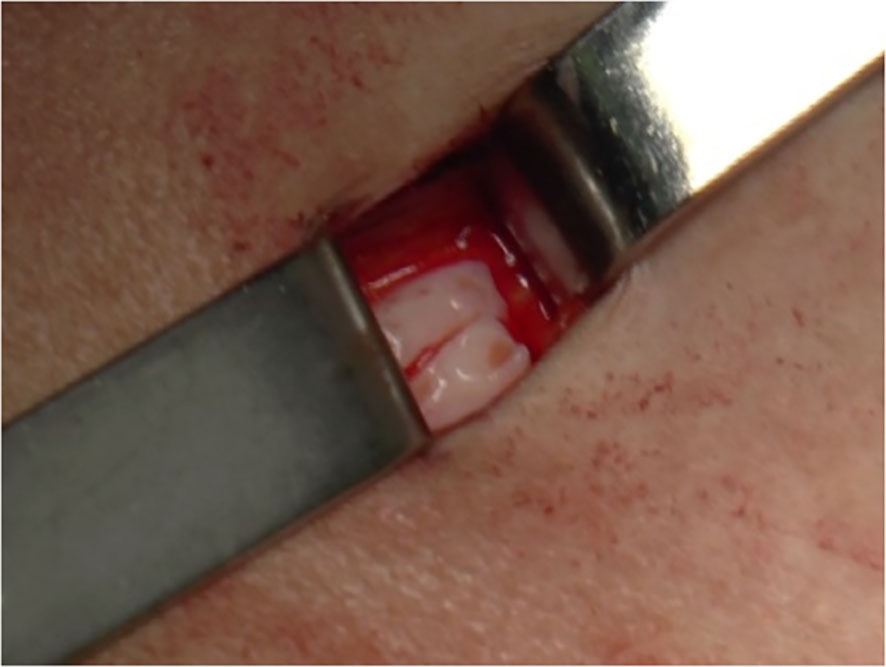
Surgical techniques of heterotopic transplantation of cryopreserved ovarian tissue. Cortical strips (sample size: 1 cm × 4 mm × 1 mm) were placed in subcutaneous pockets created above the fascia muscularis in the suprapubic area and closed with a 4-0 Vicryl suture.

For heterotopic transplantation the cortical strips were gently placed in subcutaneous pockets created above the fascia muscularis in the suprapubic area and then closed with a 4-0 Vicryl suture (**Figure 3**).

The choice of the amount of transferred tissue was evaluated for each clinical case: in most cases about 50% of the tissue is transferred during the first OTT, while more tissue is required if the women has an advanced age at the time of OTC and/or had a low ovarian reserve at the time of OTC.

### Follow-up after transplantation

After transplantation, ovarian function was monitored using hormonal assay and gynecological ultrasound at approximately 4-week intervals, until the first menstrual cycle, after which checks were scheduled based on the patients’ needs. All women with loss of ovarian activity before the age of 40 years, oligo/amenorrhea for at least 4 months, and an elevated FSH level >25 IU/l on two occasions >4 weeks apart, were considered in POI, according to European Society of Human Reproduction and Embryology (ESHRE) criteria (28).

All serum measurements were performed at the Central Laboratory of our center. FSH, LH, estradiol (E2), progesterone (P) and testosterone (T) levels were assayed using an electrochemiluminescent method (Modular; Roche), while those of antimullerian hormone (AMH) were determined by ELISA using an AMH Gen II ELISA kit (Beckman Coulter Inc., CA, USA).

All subjects underwent ultrasound examination of the internal genitalia using a multifrequency transvaginal transducer RIC5–9H (Voluson 730 Expert Sonography System; GE Healthcare Ultrasound, Zipf, Austria). Endometrial thickness, ovarian volume, number and maximum diameter of the follicles were cyclically recorded.

To examine the heterotopic graft, a high resolution volumetric linear transducer (RSP-16 multi-frequency 4D linear array transducer, Voluson 730 Expert Sonography System; GE Healthcare Ultrasound) was used. Moreover the overall ovarian tissue volume and the number and maximum diameter of the follicles were recorded.

Psychological support was offered to all women undergoing OTT during the gynecological follow-up with the specialized staff of our center.

## Results

### Subjects

Between December 2012 and June 2023, 41 women requested OTT and 30 had the procedure performed (**Table 1**). In this cohort of 30 women, the mean ± standard deviation (SD) age was 28.7 ± 6.9 years (range, 13–36) at the time of OTC. Twenty-two women transferred the cryopreserved ovarian tissue once, six women transferred twice and two women three times, with an average one-year interval between procedures. The second and third procedure was performed in cases of poor ovarian function recovery after grafting. The mean ± SD age was: 36.6 ± 5.8 (range 23-44 years) at the time of the first OTT, 38.0 ± 5.4 (range 29-45 years) at the second and 41.5 ± 0.7 (range 40-41 years) at the third.

**Table 1:** Main characteristics of women undergoing OTT (I Part).

N.	Pathology	OTC Age	Treatment before OTC	OTT Age	Storage time (years)	Number of fragments transplanted	Fresh follicular density	Frozen/thawed follicular density	Transplantation site
1	Colorectal cancer	23	NO	31	8	20/40	10.2	9.1	OT
2	Hodgkin lymphoma	29	NO	40	11	10 + 13/23	8.7	8.6	HT
3	Breast cancer	34	NO	41	7	36/36	3.8	3.7	OT
4	Myeloma	29	NO	41	12	19/19	8.1	8.3	OT
5	Struma ovarii	24	NO	35	11	15 + 10/25	10.5	9.7	OT
6	Breast cancer	37	NO	44	7	10/10	1	1.1	OT+HT
7	Breast cancer	35	NO	40	5	4 + 8/12	1.9	2.1	OT
8	Non- Hodgkin lymphoma	29	NO	34	5	4 + 6+12/27	7.8	6.4	OT
9	Hodgkin lymphoma	21	NO	27	6	4 + 5/9	16.3	15.1	OT
10	Breast cancer	33	NO	44	11	4 + 29/33	2.8	2.5	OT
11	Medulloblastoma	24	NO	31	7	2/12	8.7	8.3	OT
12	Ewing sarcoma	23	6 cycles VIDE 5 cycles VAI	31	8	4/8	11.8	10.7	OT
13	Breast cancer	36	NO	41	5	4/8	1.3	1.1	OT
14	Colorectal cancer	36	NO	39	3	5 + 10 + 8/23	1.5	1.4	OT+HT
15	Ewing sarcoma	14	NO	23	9	2/14	24.2	21.9	HT
16	Non- Hodgkin lymphoma	32	2 cycles RITUXIMAB+CHOP	35	3	8 + 7/15	5.4	5.5	OT+HT
17	Breast cancer	35	NO	43	8	22/22	1.7	1.9	OT
18	Breast cancer	32	NO	42	10	11/11	6.8	5.7	OT
19	Hodgkin lymphoma	19	6 cycles ABVD	31	11	4/18	18.4	17.2	HT
20	Uterine cervix cancer	35	NO	37	2	6/12	2.5	2	HT
21	Hodgkin lymphoma	13	6 cycles COPP-ABVD	31	18	23/56	24.3	22.5	OT
22	Non- Hodgkin lymphoma	33	NO	42	9	10/10	5.3	5.1	OT
23	Breast cancer	34	NO	41	7	10/22	5.6	4.2	OT
24	Breast cancer	35	NO	43	8	11/28	1.5	1.2	OT
25	Ewing sarcoma	22	1 cycle: ifosfamide, adriamicina, vincristina, 1 cycle: ifosfamide, etoposide	35	13	13/13	15.3	15.1	OT
26	Breast cancer	30	NO	39	9	14/14	9.1	8.7	OT
27	Non- Hodgkin lymphoma	20	NO	31	11	8/15	18.2	14.5	OT
28	Breast cancer	32	NO	40	8	11/23	4.2	3.8	OT
29	Breast cancer	33	NO	43	10	13/25	5	4.7	OT
30	Hodgkin lymphoma	13	NO	30	17	7/15	35	28	OT
**MEAN**		**28.2**		**36.8**	**8.6**		**9.5**	**8.3**	

N, number; OTT, Ovarian tissue transplantation; OT, Orthotopic transplant; HT, heterotopic transplant; VIDE, vincristine, ifosfamide, doxorubicin, etoposide; VAI, vincristine, actinomycin, ifosfamide; CHOP, doxorubicin, cyclophosphamide,vincristine, prednisone; ABVD, doxorubicin, bleomycin, vinblastine, dacarbazine; COPP-ABVD, cyclophosphamide, vincristine, procarbazine, prednisone, doxorubicin, bleomycin, vinblastine, dacarbazine; Follicular density, number of follicles per mm^2^.

At the first OTT, 66.7% of women (20 cases) were on POI, and the remaining 33.3% (10 cases) still had oligomenorrhea and difficulty getting pregnant so it was decided to increase their ovarian reserve with additional OTT.

OTT was not carried out in eleven women for the following reasons: in four cases for ovarian tissue contamination (two Non-Hodgkin lymphomas detected by immunohistochemistry, one acute myeloid leukemia and one chronic myeloid leukemia detected by specific molecular analysis); in three cases due to lack of approval by the oncologist/hematologist (one myelofibrosis, one myelodysplastic syndrome and one acute lymphoblastic leukemia); in two cases for contraindication to laparoscopy (one anemia and one adenocarcinoma of the uterine cervix) and in two cases for personal choice due to fear of surgery (one myeloma and one acute myeloid leukemia).

### Pre-OTC diagnosis

All women who performed OTT had cryopreserved tissue for a cancer diagnosis. Breast cancer accounted for 40% (12 cases) of women, while hematologic cancers accounted for 30% (nine cases), including five Hodgkin and four non-Hodgkin lymphomas. Other malignancies in decreasing order of frequency were: Ewing sarcoma (10%, three cases), colonrectal cancer (6.8%, two cases), myeloma (3.3%, one case), medulloblastoma (3.3%, one case), uterine cervix cancer (3.3%, one case) and struma ovarii (3.3%, one case).

Pre-OTC chemotherapy treatments were performed in two women affected by Ewing sarcoma, two affected by Hodgkin lymphoma and one affected by non-Hodgkin lymphoma as reported in **Table 1**. Pre-OTT radiotherapy treatments (total body irradiation - TBI - and pelvic irradiation - PI) were performed in six patients with following diseases: Medulloblastoma (1- TBI), Ewing sarcoma (2 PIs), colorectal cancer (2 PIs) and uterine cervix cancer (1 PI). All patients recovered endocrine function after orthotopic OTT and one of them (Ewing sarcoma) achieved a live birth (**Table 2**).

**Table 2:** Main outcomes in women undergoing OTT.

N.	Pathology	Recovery time of menstrual cycles after 1^st^ OTT (months)	Ovarian function duration (months)	Spontaneous pregnancy	IVF pregnancy	Babies born
1	Colorectal cancer	4	24, Ceased			
2	Hodgkin Lymphoma	6	84, Ceased			
3	Breast cancer	4	7, Ceased			
4	Myeloma	4	12, Ceased			
5	Struma Ovarii	None	/			
6	Breast cancer	None	/			
7	Breast cancer	Oligomenorrhea	72, Still present			
8	Non- Hodgkin Lymphoma	5	84, Still present	1		1
9	Hodgkin Lymphoma	5	84, Still present		1	1
10	Breast cancer	Oligomenorrhea	62, Still present			
11	Medulloblastoma	3	24, Started therapy			
12	Ewing sarcoma	3	60, Still present	1		1
13	Breast cancer	Oligomenorrhea	70, Still present	3		1
14	Colorectal cancer	6	22, Ceased			
15	Ewing sarcoma	4	24, Started therapy			
16	Non- Hodgkin Lymphoma	4	20, Ceased			
17	Breast cancer	Oligomenorrhea	Lost to follow-up			
18	Breast cancer	Oligomenorrhea	Lost to follow-up			
19	Hodgkin Lymphoma	4	26, Still present			
20	Uterine cervix Cancer	4	22, Ceased			
21	Hodgkin Lymphoma	4	26, Still present			
22	Non- Hodgkin Lymphoma	4	8, Ceased			
23	Breast cancer	Oligomenorrhea	24, Still present			
24	Breast cancer	Oligomenorrhea	22, Still present			
25	Ewing sarcoma	4	22, Still present	1	1 (ongoing)	
26	Breast cancer	Oligomenorrhea	20, Still present	1	1 (ongoing)	
27	Non- Hodgkin Lymphoma	4	19, Still present			
28	Breast cancer	Oligomenorrhea	20, Still present			
29	Breast cancer	Oligomenorrhea	18, Still present			
30	Hodgkin Lymphoma	4	10, Still present			
**TOTAL**				**7**	**3**	**4**

N, number; OTT, Ovarian tissue transplantation; IVF, in vitro fertilization.

### Analysis of fresh and thawed ovarian tissue

Homogeneous distribution of stromal cells with regular nuclei was observed in all fresh and thawed samples. Mild interstitial edema, widespread vacuolization and marked chromatin clumping were found in thawed samples. In both fresh and thawed samples, most follicles were in the resting stage, while only a few follicles were in the growing stage. The percentage of morphologically normal follicles was not significantly different between fresh (91.6 ± 8.2%) and thawed tissue (83.5 ± 12.8%). After thawing, the alterated follicles showed oocytes with empty-looking cytoplasm, in which no mitochondria were distinguishable. This alteration was also present in damaged fresh follicles.

The mean ± SD follicular density (number of follicles per mm^2^ of the overall section area) in fresh and thawed samples was 9.5 ± 7.1 follicles/mm^2^ (range 1-35 follicles/mm^2^) and 8.3 ± 6.2 follicles/mm^2^ (range 1.1-28 follicles/mm^2^), respectively.

**Figure 4:**
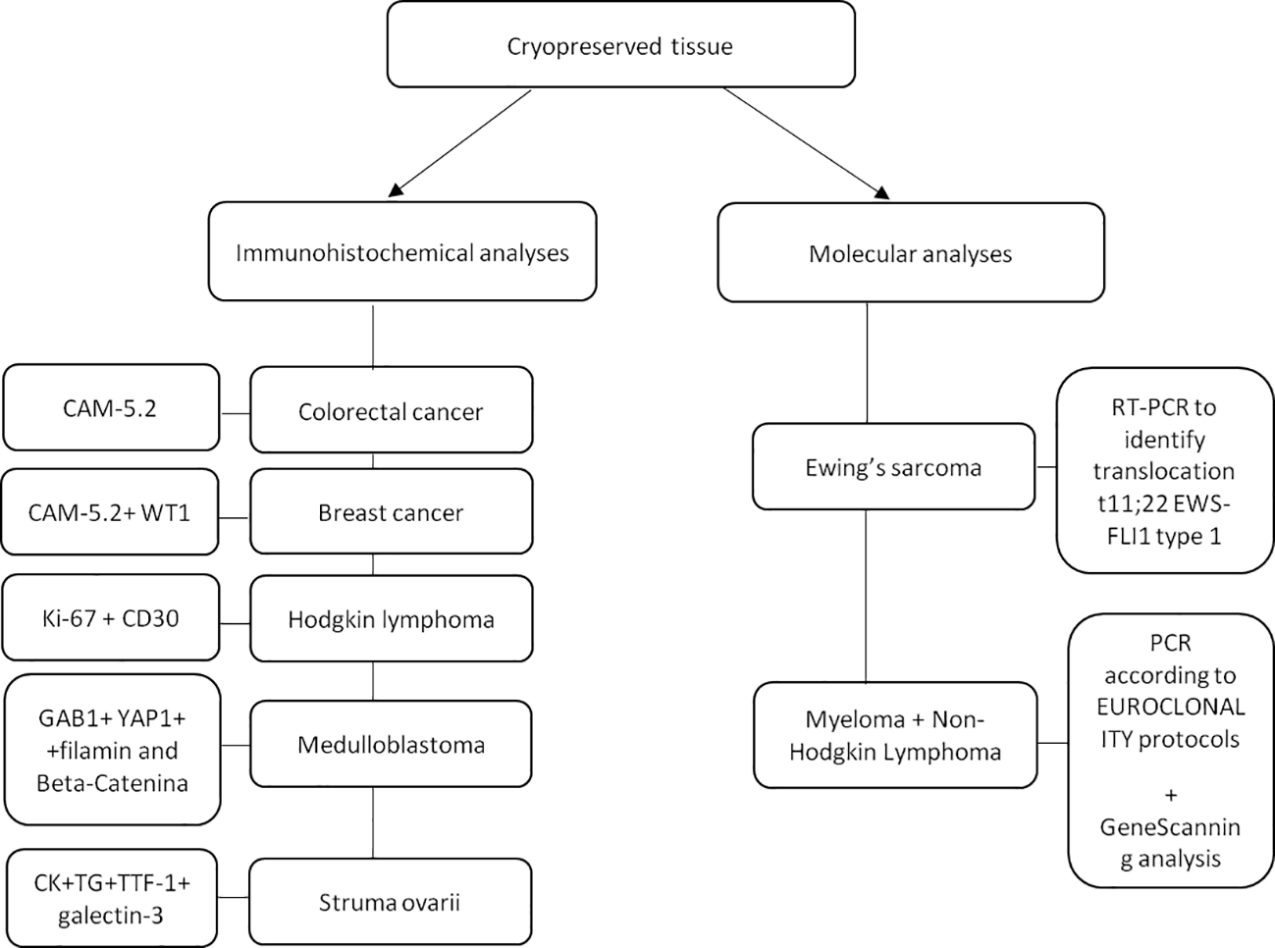
Patient’s specific immunohistochemical and molecular analyses performed on a small fragment of ovarian tissue to detect the presence of micrometastases. CAM-5.2: Cytokeratin; WT1: Wilms tumor-suppressor gene-1; Gab1: GRB2-associated-binding protein 1; Yap1: yes-associated protein 1; CK: creatinchinasi; TG: Thyroglobulin; TTF1: Thyroid transcription factor-1; PCR: Polymerase chain reaction.

Prior to OTT, immunohistochemical and molecular analyses revealed no micrometastases in all thawed ovarian tissue samples. In particular, the following monoclonal antibodies were used for immunohistochemical analysis: cytokeratin CAM-5.2 (1:20; Becton Dickinson, CA, USA) to detect cells originating from colorectal cancer; cytokeratin CAM-5.2 and WT1 (1:50; Novocastra, Newcastle Upon Tyne, UK) to detect breast cancer cells; GAB1, YAP1, filamin and Beta-Catenina to detect medulloblastoma cells; Ki-67 (1:230; Novocastra™, Newcastle upon Tyne, UK) and CD30 (1:40; Dako, Glostrup, Denmark) to detect Reed–Sternberg cells in Hodgkin lymphoma; cytokeratin, thyroglobulin, thyroid transcription factor 1 and galectin-3 to detect struma ovarii in ovarian tissue. For women with Ewing’s sarcoma, the presence of ovarian micrometastases was evaluated by Real-Time RT-PCR to identify the specific translocation t11;22 EWS-FLI1 type 1. For women with myeloma and non-Hodgkin lymphoma, the PCR assay was performed according to EUROCLONALITY protocols (29) and evaluated by GeneScanning analysis to determine the rearrangement of the genes coding for heavy immunoglobulins (IGH; FR1-FR2-FR3 regions) and light chains (IGK), since no other patient-specific markers were available (**Figure 4**).

### Ovarian tissue transplantation

**Figure 5:**
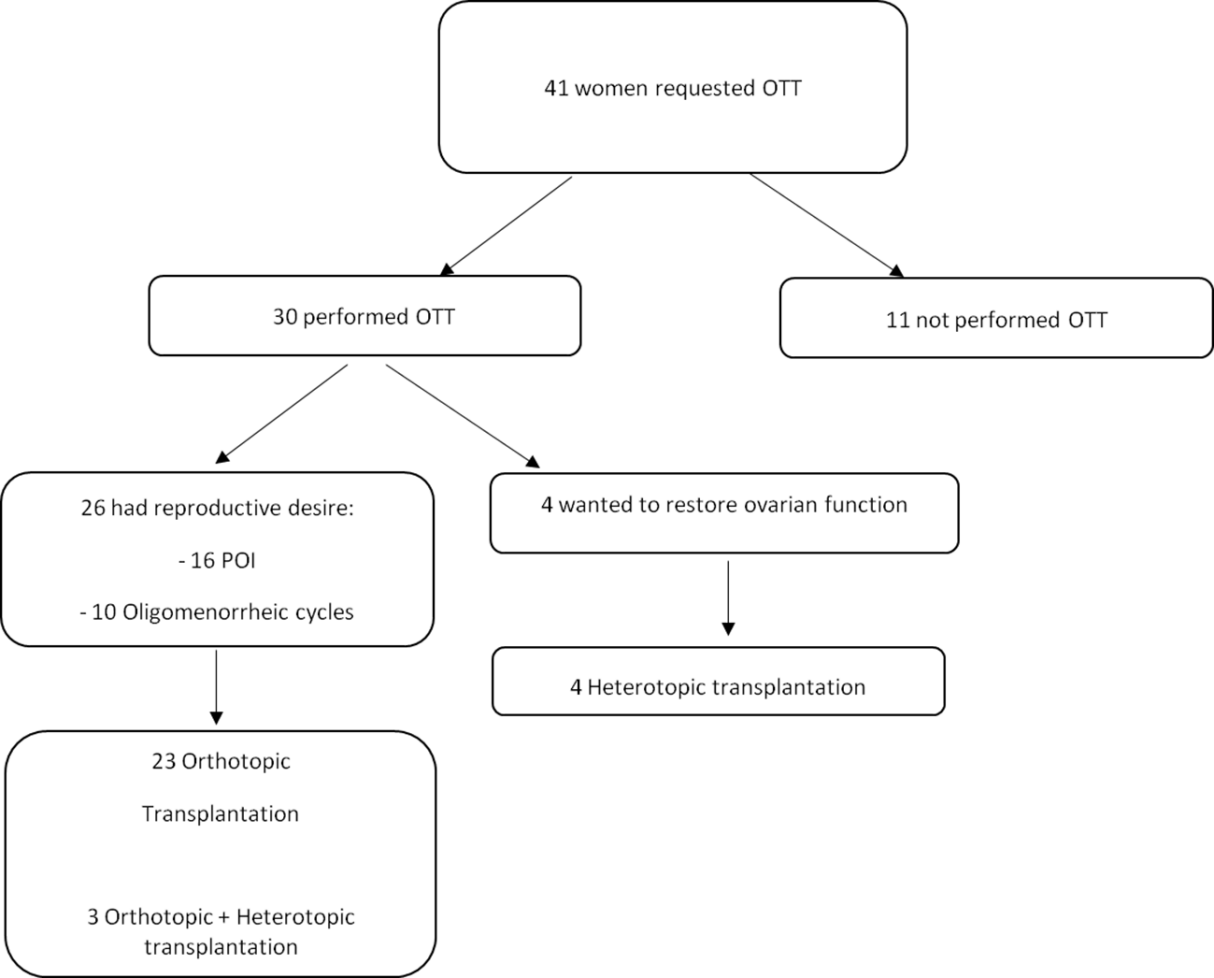
Women who request OTT between December 2012 and June 2023 at our center.

In our series of 30 women, 23 (76.7%) underwent orthotopic transplantation for reproductive desire, three (10%) underwent both orthotopic and heterotopic transplantation for reproductive desire, and four (13.3%) underwent heterotopic transplantation only to restore ovarian function (**Figure 5**). Among those undergoing orthotopic transplantation, thawed ovarian fragments were grafted into the surface of the ovaries and into the peritoneal pocket created near the ovarian vessels and fimbria.

The mean amount of transplanted tissue (number of fragments thawed/total number of fragments stored*100) was 51.9% at the first OTT, 29.6% at the second, and 18.5% at the third. For eight women it was decided to transplant all the cryopreserved cortical slices during the first OTT because the follicular density was very low (one follicle per mm^2^). For a total of 15 women, all cryopreserved ovarian tissue samples were thawed for transplantation, while 15 women still have cryopreserved ovarian tissue for further OTT. No perioperative complications were recorded in all women.

**Table 1** shows pre-OTC diagnosis of the women undergoing OTT, their age at the time of cryopreservation and transplantation, site of transplantation and amount of tissue transplanted.

### Restoration of endocrine function and longevity

The recovery of endocrine function was evaluated in all women with POI at the time of OTT after at least 6 months follow-up. Endocrine function was measured using hormonal assays, follicular growth and recurrence of menstruations. Among women with POI before OTT and at least 6 months follow-up (20 cases), recovery of endocrine function was observed in all out of one woman who underwent orthotopic transplantation (92.9%, 13 of 14 cases), in one out of two women who underwent both orthotopic and heterotopic transplantation (50%, 1 of 2 cases) and in all women who underwent heterotopic transplantation (100%, 4 of 4 cases). About three months after the first OTT, women reported cessation of menopausal symptoms, the presence of breast tenderness and the presence of preovulatory mucus. FSH and LH levels decreased, and estradiol levels increased (**Table 3**). Subsequently, follicular development was observed at the graft sites using ultrasound evaluation (**Table 3**). Women had their first spontaneous mense 4.2 ± 0.8 months after OTT (**Table 2**). Recovery of endocrine function after the second and third OTT was observed approximately two months after the surgery.

**Table 3:** The average concentrations of hormonal levels and mean number of antral follicles before OTT (Pre-OTT) and at the time of first menstrual cycle after OTT (Post-OTT).

	*N*	*Pre-OTT*							*Post-OTT*						
		*FSH* *	*LH* *	*E2* *	*P* *	*T* *	*AMH* *	*FOL. N.*	*FSH* *	*LH* *	*E2* *	*P* *	*T* *	*AMH* *	*FOL. N.*
***POI***	*20*	110.6	58.9	20	0.15	0.34	0.02	0	2.6	6.0	250	11.70	0.29	0.03	5
***No-POI***	*10*	1.8	5.3	285	16.75	0.38	1.24	5	3.1	5.5	263	17.82	0.35	1.0	7

POI, premature ovarian insufficiency; N, number; FSH, Follicle-stimulating Hormone; LH, luteinizing hormone; E2, estradiol; P, progesterone; T, testosterone; AMH, Anti-mullerian hormone; Fol. N., Follicle number.

*FSH mU/mL, LH mU/mL, E2: pg/mL, P ng/mL, T ng/mL, AMH ng/Ml.

All women who underwent OTT to enhance fertility, had regular menstrual cycles and hormonal levels for the patient’s age (100%, 10 of 10 cases). Approximately, three months after OTT, an increase in antral follicles was observed.

The lifetime of ovarian activity was evaluated in a subgroup of twenty-six women with POI or oligomenorrhea before OTT, with complete hormonal follow-up information (at least six months). The outcomes showed a lifetime of ovarian activity ranging from 0 to 84 months with a mean of 34.1 months, regardless of the woman’s age and previous chemotherapy before OTC. The longest period of ovarian activity obtained after OTT was 84 months in a case of Hodgkin lymphoma who underwent heterotopic OTT twice (**Table 2**).

### Pregnancies and live births

The number of pregnancies and live births was evaluated in all OTT women after at least 1 year follow-up (**Table 2**). Out of a total of 30 women, 26 wished to restore and improve fertility: 16 were on POI before OTT and 10 had oligomenorrhea.

Among all OTT women after at least 1 year follow-up, ten (38.5%) pregnancies were obtained in 26 women, resulting in four live births, two ongoing pregnancy and four spontaneous abortions. Out of the four live births, three were obtained in POI women healed from non-Hodgkin lymphoma, Hodgkin lymphoma and Ewing Sarcoma, respectively. The last live birth and three abortions were obtained in two women healed from breast cancer and with oligomenorrhea at the time of OTT. The last abortion was obtained in a woman healed from Ewing Sarcoma in POI at the time of OTT. Ongoing pregnancies were obtained in two women healed from Ewing Sarcoma and breast cancer, respectively.

*In vitro* fertilization (IVF) was proposed to women after one year of unsuccessful pregnancy attempts. Of the ten pregnancies, one live birth and two ongoing pregnancies (30%) were obtained with IVF, while all the others (70%) were spontaneous pregnancies.

### Recurrence

All 30 women who performed OTT had tissue cryopreserved for a cancer diagnosis. Of the whole group, only one woman had a recurrence after OTT. This is a woman diagnosed with sacral Ewing sarcoma who had a recurrence localized to the shoulder five months after OTT. The recurrence was considered unrelated to OTT and dependent on the primary disease. The woman is among those who became pregnant and delivered after OTT, and to date, four years later she is in good health and disease free.

## Discussion

This study reports the outcomes obtained over 10-years experience with the OTT technique in the restoration of fertility and ovarian function in cancer women. Our data showed a transplantation rate of approximately 2% (30), ovarian function recovery rate about 93%, pregnancies rate of 40% and take-home baby rate of around 16%. Our data are in agreement with those reported by Khattak et al. (16), that performed a meta-analysis of twenty studies reported on ≥5 cases of ovarian transplants (568 women). Fertility outcomes included pregnancy, live birth, miscarriage rates, and endocrine outcomes included estrogen, FSH and LH levels. The pooled rates were 37% (95% CI: 32–43%) for pregnancy, 28% (95% CI: 24–34%) for live birth and 37% (95% CI: 30–46%) for miscarriage following frozen ovarian tissue transplantation. Also the hormones data show evidence of return of hormonal function after transplantation with significant decrease in FSH and LH, and an increase in estrogen post ovarian transplant, in accordance with the major achievements (16). AMH does not undergo substantial changes, turning out not to be an indicator that can help in understanding the state of ovarian activity post-OTT.

There are several reasons why OTT was performed in a low number of women. Despite having over a thousand ovarian tissue’s cryobanked, a marked increase for OTT was registered only in the recent years. Oncologists recommend at least five years between chemotherapy and transplantation and, very often, the women wait even further before requesting surgery (1, 30). In addition, many women did not experience POI after cancer therapy, some died of cancer, and some, especially prepubertal girls, were still too young to attempt pregnancy at the end of their cancer follow-up.

The effectiveness of this technique has already been recognized in the literature (1, 2) as ovarian function recovery (95% of cases), number of live births exceeding 200 and induction of puberty (10–12).

It is very important to focus on the high recovery rates of endocrine function after OTT. As we reported (31), heterotopic transplantation could be a useful tool to eliminate menopausal symptoms without causing or aggravating other health risks. In addition, with a broader clinical perspective, heterotopic transplantation could potentially save enormous health care costs, preventing osteoporosis and reducing cardiovascular risks. Ovarian function recovery expands the traditional fertility purposes of OTC, among which the possibility to use OTC for cell/tissue-based hormone replacement therapy (32).

Graft success can be influenced by several factors including age at cryopreservation, type of cancer treatment, freezing-thawing protocols, transplantation techniques and graft sites (33). All these factors were analyzed despite the relatively small number of cases studied and regardless of the heterogeneity of these women with multiple factors affecting outcomes.

Women’s age at the time of OTC is an important predictive factor of ovarian graft success. The younger the women are at the time of OTC, the greater is the potential for recovery of endocrine function after OTT, given the larger baseline density of primordial follicles. It seems that 35 years of age is considered as a limit for cryopreservation techniques, since the number of primordial follicles decreases significantly after this age (34). In our center, the age limit has been set at 38 years, albeit with some limitations for women between 35 and 38, who undergo thorough screening of the ovarian reserve (AMH levels and sonographic antral follicle count) before proceeding to OTC (35).

Of the 30 women who underwent OTT, only five were treated with chemotherapy before OTC and one of these had a birth. Chemotherapy can lead to qualitative alterations of follicles and oocytes, as well as vascular damage, resulting in ovary fibrosis (1). For this reason, it is recommended to perform OTC before starting chemotherapy (1). Recent guidelines from European Society of Human Reproduction and Embryology (ESHRE) indicate that ‘‘patients who have already received low gonadotoxic treatment or a previous course of chemotherapy can be offered OTC as a fertility preservation option’’ (36). Dolmans et al. (1), confirmed that there was no difference in the recovery rate of ovarian function in patients treated or not with chemotherapy before OTC and that pregnancies and live birth rates were higher in patients treated with chemotherapy before OTC (1). No specific conclusions were drawn by us, due to the low number of women studied.

In our cohort of 30 women, 6 received some kind of irradiation before OTT. According to literature results ovarian tissue can be transplanted after pelvic radiotherapy if the radiation exposure was relatively low, as in the case of TBI (1). However, because of the poor success rates and obstetric risks associated with diseases requiring high pelvic radiation doses, such as anal, rectal, and cervical cancer, OTC and OTT should be carefully considered before going ahead. Data from literature demonstrated that ovarian tissue can be transplanted after radiation, but probably only according to specific criteria, taking into account not only the disease, but also the radiation zone and dose (1). No specific conclusions were drawn by us, due to the low number of women studied.

Another key factor is the standardization of cryopreservation procedures. Our protocol was optimized over the years by the comparison of fresh and frozen/thawed tissue after short-term storage as reported by us (21, 37). Of course, only the recovery of ovarian function after OTT is able to demonstrate the true capabilities of cryopreserved and stored ovarian tissue (37).

Of all the existing surgical techniques for OTT, orthotopic reimplantation has proved to be the most effective in both recovery of endocrine function and restoration of fertility (1). There are two important observations. First, successful OTT approaches should adhere to fundamental microsurgical principles, namely, finding a well-vascularized graft site and a way to affix the ovarian tissue. Second, the amount of transplantation tissue must be carefully chosen, anticipating the potential need for further reimplantation in the same patient. It is recommended to graft only a portion of the cryopreserved tissue, at least when a large amount of tissue was initially cryopreserved (as in the case of oophorectomy or large bilateral biopsies) (1).

The safety of the procedure is a pivotal aspect when planning transplant; it is well known that there are risks of re‐introducing cancer cells from transplanted ovarian tissues. The potential of ovarian metastasis has been categorized based on systematic studies (38). Based on the literature review, histology has been validated for many years and the use of immunohistochemistry (IHC) is also standardized. However, the use of advanced and highly sensitive molecular approaches (RT-PCR) can greatly improve the detection rate of malignant cells in ovarian tissue. Once specific and predominant gene mutations or chromosomal rearrangements have been searched in the primary tumor, they can serve as tumor-related markers for the detection of malignant cell infiltration in the ovarian tissue. These analyses can only confirm the presence of the malignant cells, but they cannot give informations about the viability or the malignant potential of the cells themself if transplanted. Moreover, these procedures (IHC and RT‐PCR) destroy the examined tissues that can no longer be used for OTT. Finally, it is unclear how many malignant cells are needed to cause cancer to recur; it may vary among individuals and malignancies (39). Lacking universal consensus on this topic we decided to systematically perform safety tests before OTT per center policy. In our series histological, immunohistochemical and molecular investigations on ovarian tissues showed no evidence of pathological infiltration. Transplantation in women diagnosed with previous malignancy was performed 30 times and there was only one case of recurrence due to the primary disease and not related to OTT (26).

Despite the encouraging data, contemporary studies are focusing on developing new techniques that can improve the efficiency of OTT. Roness et al. (40) conducted a study evaluating current approaches to maximize graft lifespan via pharmacological agents, such as angiogenic, antioxidant, anti-apoptotic and anti-inflammatory agents. Revascularization and prevention of apoptosis are key factors in ensuring the survival of the ovarian tissue graft, since ischemia and hypoxia have been reported as the main obstacles to successful OTT. Furthermore, the reduction of follicle activation has the potential to reduce follicle loss after transplantation (39, 40).

## Conclusion

The field of fertility preservation is constantly evolving. There is an ethical obligation to provide information on the impact of cancer treatment on future fertility and to discuss fertility issues with patients. Our data can help women and physicians in their discussions and decisions about the need and possibilities of preserving fertility.

## Data availability statement

The raw data supporting the conclusions of this article will be made available by the authors, without undue reservation.

## Ethics statement

The study involving humans were approved by Division of Gynaecology and Human Reproduction Physiopathology, IRCCS Azienda Ospedaliero-Universitaria di Bologna, Bologna, Italy N. 74/2001/O. The study were conducted in accordance with the local legislation and institutional requirements. The participants provided their written informed consent to participate in this study.

## Author contributions

RF: Data curation, Methodology, Supervision, Writing – original draft, Writing – review & editing, Formal analysis, Conceptualization, Investigation, Validation. RV: Data curation, Formal analysis, Methodology, Supervision, Writing – original draft, Writing – review & editing, Investigation. VM: Data curation, Formal analysis, Methodology, Supervision, Writing – original draft, Writing – review & editing, Investigation. RP: Data curation, Formal analysis, Methodology, Supervision, Writing – original draft, Writing – review & editing, Investigation. LDM: Data curation, Formal analysis, Methodology, Supervision, Writing – review & editing, Investigation. DR: Data curation, Formal analysis, Methodology, Supervision, Writing – review & editing, Investigation. AA: Data curation, Formal analysis, Methodology, Supervision, Writing – review & editing, Investigation. SV: Data curation, Formal analysis, Methodology, Supervision, Writing – review & editing, Conceptualization, Investigation. ARaf: Data curation, Formal analysis, Writing – original draft, Writing – review & editing. ARas: Data curation, Formal analysis, Writing – original draft, Writing – review & editing. RS: Data curation, Formal analysis, Methodology, Supervision, Writing – review & editing, Conceptualization, Investigation.
